# The Impact of Fetal Growth Restriction on Prenatal 2D Ultrasound and Doppler Study of the Fetal Adrenal Gland

**DOI:** 10.1155/2024/9968509

**Published:** 2024-08-29

**Authors:** Suphawan Pattamathamakul, Chatuporn Duangkum, Sukanya Chaiyarach, Kiattisak Kongwattanakul, Piyamas Saksiriwuttho, Ratana Komwilaisak, Sathida Chantanavilai, Manasicha Pongsamakthai, Prapassara Sirikarn

**Affiliations:** ^1^ Department of Obstetrics and Gynecology Faculty of Medicine Khon Kaen University, Khon Kaen, Thailand; ^2^ Department of Obstetrics and Gynecology Khon Kaen Hospital, Khon Kaen, Thailand; ^3^ Department of Epidemiology and Biostatistics Faculty of Public Health Khon Kaen University, Khon Kaen, Thailand

**Keywords:** adrenal artery, fetal adrenal gland, fetal growth restriction, inferior adrenal artery, neocortex

## Abstract

**Background:** Uteroplacental insufficiency in fetuses with growth restriction (FGR) leads to chronic hypoxia and stress, predominantly affecting the adrenal glands. However, the mechanisms of impact remain unclear.

**Objectives:** This study is aimed at comparing the Doppler indices of the adrenal artery and the adrenal gland sizes between FGR and those with normal growth.

**Materials and Methods:** A multicenter, cross-sectional study was conducted from February to December 2023. We compared 34 FGR to 34 with normal growth in terms of inferior adrenal artery (IAA) Doppler indices and adrenal gland volumes.

**Results:** The IAA peak systolic velocity (PSV) in the FGR group was 14.9 ± 2.9 cm/s compared to 13.5 ± 2.0 cm/s in the normal group, with a mean difference of 1.4 cm/s (95% confidence interval [CI]: 0.27–2.65; *p* value = 0.017). There were no significant differences between groups in terms of IAA pulsatility index (PI), resistance index (RI), or systolic/diastolic (S/D), with *p* values of 0.438, 0.441, and 0.658, respectively. The volumes of the corrected whole adrenal gland and the corrected neocortex were significantly larger in the FGR group, with *p* values of 0.031 and 0.020, respectively.

**Conclusion:** Both increased IAA PSV and enlarged volumes of the corrected whole adrenal gland and neocortex were found in fetuses with FGR, suggesting significant adrenal gland adaptation in response to chronic intrauterine stress.

## 1. Introduction

Fetal growth restriction (FGR) is defined as a pathological in utero growth disorder primarily caused by factors related to the fetus, the mother, or the placenta [1–3]. Neonatal mortality rates are higher in FGR compared to cases in which there is normal growth [1, 2]. Additionally, FGR is associated with increased risks of both short- and long-term neonatal morbidities, such as intraventricular hemorrhage, infections, respiratory distress, delayed brain development, impaired endocrine function, and cardiovascular disease [1–9]. The principal cause of placenta-related FGR is insufficient remodeling of the uterine spiral arteries that supply the placenta [10–13]. This inadequate arterial remodeling leads to compromised blood flow, which stresses placental cells, ultimately decreasing the placental villi's volume and surface area available for maternal–fetal nutrient and waste exchange. Consequently, this reduction in villous volume and surface area results in chronic fetal hypoxia and stress [9, 12–14]. The maintenance of blood supply to vital organs such as the brain, myocardium, and adrenal glands requires redistribution of fetal circulation, primarily through the hypothalamus-pituitary-adrenal axis (HPA) [1, 9, 15, 16]. Glucocorticoid (GC) hormones, particularly cortisol, are crucial in managing stress responses during fetal development and in regulating the growth and maturation of fetal tissues and organs [17, 18]. In contrast, studies of placental vascular diseases associated with FGR have reported elevated plasma cortisol levels and decreased levels of adrenocorticotropic hormone (ACTH) in affected fetuses compared to those with normal growth [19].

The fetal adrenal glands, appearing early at 28–30 days postfertilization, are among the largest organs when the fetus is near term [20]. The fetal adrenal cortex undergoes rapid growth during the prenatal period and divides into three zones: the fetal zone (FZ), the definitive zone (DZ), and the transitional zone (TZ). The fetal adrenal medulla, however, is not recognizable until delivery [21]. The FZ is responsible for the synthesis of dehydroepiandrosterone (DHEA) and dehydroepiandrosterone sulfate (DHEA-S), which are crucial for facilitating placental estrogen production. Meanwhile, the DZ and TZ, also known as the “neocortex,” are involved in the production of cortisol and aldosterone during pregnancy [20–25].

The fetal adrenal glands comprise a highly vascularized organ, which receives blood from several primary arteries: the superior adrenal artery (SAA), middle adrenal artery (MAA), and inferior adrenal artery (IAA), which originate from the inferior phrenic artery, abdominal aorta, and renal artery, respectively [20, 26]. The superior and inferior portions of the DZ are primarily supplied by the SAA and IAA, respectively, while the FZ is predominantly supplied by the MAA [26, 27].

Theoretically, chronic fetal hypoxia and stress could trigger the activation of the HPA axis, potentially affecting both the adrenal vessels and the adrenal glands [15, 25, 28, 29]. Therefore, the aim of this study is to compare the differences in Doppler indices of the adrenal artery and adrenal gland sizes between fetuses with growth restriction and those with normal growth.

## 2. Materials and Methods

This was a multicenter cross-sectional study conducted at two participating tertiary hospitals in Thailand: Srinagarind Hospital and Khon Kaen Hospital, between February and December 2023. The study was approved by the ethics committees of both sites, the KKU Ethics Committee in Human Research (HE651571) and the Khon Kaen Institute Review Board in Human Research (KEMOU66007). The study also adhered to the STROBE guidelines.

### 2.1. Inclusion Criteria

The inclusion criteria were singleton pregnancy and age 18 years or older, with gestational age (GA) between 24 and 40 weeks. The study group included fetuses with growth restriction due to uteroplacental insufficiency (UPI), and the control group consisted of fetuses with normal growth.

### 2.2. Exclusion Criteria

The exclusion criteria included prepregnancy body mass index (BMI) of 30 kg/m^2^ or more, underlying adrenal gland disease, having received steroid treatment, and obstetric complications such as preterm labor or antepartum hemorrhage in the current pregnancy. Women were also excluded if their fetuses had growth restriction due to causes other than UPI, including fetal anomaly, abnormal karyotype, or infection, or if they could not be assessed by either Doppler indices or adrenal gland size.

### 2.3. Outcomes

The primary outcome was the difference in Doppler indices of the adrenal artery between the two groups. The secondary outcome was the difference in adrenal gland sizes. The sample size was calculated to detect a 20% mean difference in adrenal artery pulsatility index (PI), resulting in a requirement of 68 women (34 per group) to achieve a statistical power of 80% at a significance level of 0.05.

### 2.4. Study Definitions

FGR and UPI were defined as fetal weight less than the 10th percentile for GA with a PI of the umbilical artery (UA) greater than the 95th percentile or a cerebroplacental ratio (CPR) of less than the 5th percentile, confirmed by placental histopathological evidence [30, 31]. Early-onset FGR was diagnosed before 32 weeks of GA, and late-onset FGR was diagnosed at 32 weeks of GA or later [30]. Normal growth was defined as an estimated weight between the 10th and 90th percentiles for GA [1].

### 2.5. Study Procedure

After obtaining informed consent from the participants, fetal biometric parameters were assessed. The estimated fetal weight was determined using Hadlock's formula [32]. Doppler velocimetry of the IAA, UA, and middle cerebral artery (MCA) was performed. All measurements were conducted using a Voluson E8 BT20 machine (GE Healthcare, United States) equipped with a 2–5 MHz frequency abdominal curvilinear transducer.

As a previous study reported no significant difference in Doppler indices between the right and left adrenal vessels [33], this study evaluated only the unilateral adrenal gland. The adrenal gland closest to the ultrasound beam was selected for measurement, and the fetal adrenal gland image was magnified to 75% of the screen.

**Figure 1:**
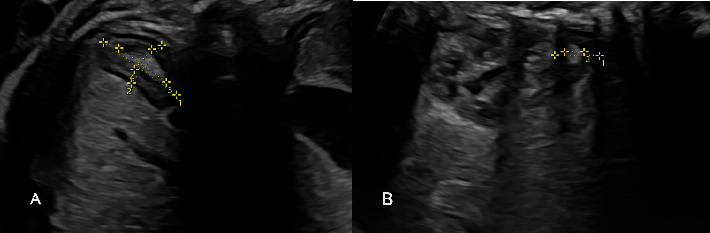
Two-dimensional fetal adrenal gland ultrasonography. (A) Axial plane: length (1) and width (2) of the WAG and length (3) and width (4) of the FZ. (B) Sagittal plane: depth of the WAG (1) and depth of the FZ (2). Abbreviations: FZ, fetal zone; WAG, whole adrenal gland.

The fetal adrenal gland was identified in the fetal abdominal scanning plane, located above the fetal kidney. The FZ appeared as a central hyperechoic area of the adrenal gland, surrounded by the neocortex, a hypoechoic area. The length and width of the whole adrenal gland (WAG) and the FZ were measured in an axial plane (Figure 1(a)), while their depth was measured in a sagittal plane (Figure 1(b)). The volume of the WAG (WAGV) and the FZ (FZV) were calculated in cubic millimeters (mm^3^) using the ellipsoid formula: volume = (*π*/6) × width (mm) × length (mm) × depth (mm).

The volume of the fetal neocortex (NV) was calculated by subtracting the FZV from the WAGV. Due to variability in adrenal gland growth across different GAs and fetal weights [34, 35], corrected volumes were used to standardize variables and minimize potential confounding factors. The corrected WAGV (C´WAGV), corrected FZV (C´FZV), and corrected NV (C´NV) were calculated by dividing the volume by the estimated fetal weight.

**Figure 2:**
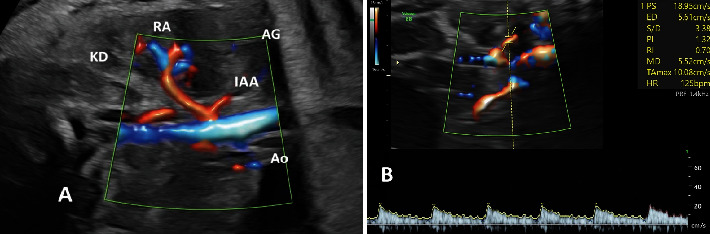
(A) Doppler color flow of IAA in oblique-coronal plane. (B) The IAA Doppler pulse waves and indices. Abbreviations: AG, adrenal gland; Ao, aorta; IAA, inferior adrenal artry; KD, kidney; RA, renal artery.

The IAA was identified in an oblique-coronal plane of the fetal abdomen, originating from the trunk of the renal artery and passing the inferoposterior border of the adrenal gland (Figure 2(a)). Spectral Doppler was applied to obtain the IAA waveform, measuring the lowest blood flow. The sample volume was adjusted between 1.5 and 2 mm, placed at the middle course of the IAA, with the angle of insonation maintained at less than 30°. At least three velocity waveforms were recorded and automatically measured during periods without fetal movement or breathing (Figure 2(b)). The IAA peak systolic velocity (PSV), PI, resistance index (RI), and the ratio of systolic to diastolic velocities (S/D) were measured in triplicate, and the average values were recorded.

The intraclass correlation coefficient (ICC) was used to evaluate the intrarater and interrater reliabilities. Reliability was classified as poor (ICC < 0.5), moderate (0.5 ≤ ICC < 0.75), good (0.75 ≤ ICC < 0.9), and excellent (ICC ≥ 0.9) [36]. The ICC was assessed from 10 participants by two sonographers (S.P. and C.D.).

Maternal characteristics, including demographic data, GA at the time of examination, and route of delivery, were recorded. Neonatal outcomes, such as GA at delivery, birth weight, gender, APGAR score, adverse outcomes, neonatal intensive care unit (NICU) admission, and length of hospital stay, were also documented.

### 2.6. Statistical Analysis

Maternal and fetal characteristics were presented as mean ± standard deviation (SD) for continuous data and frequency (percentage) for categorical data. All analyses were performed using STATA software Version 15.0 (College Station, Texas, United States). We used the Wilcoxon rank sum test model to evaluate the mean differences and 95% confidence intervals (CIs) for nonparametric data and an independent *t*-test for parametric data, adjusted by GA at the time of examination between the two groups. A sensitivity analysis compared early and late FGR to the normal group. A *p* value < 0.05 was considered statistically significant.

## 3. Results

**Figure 3:**
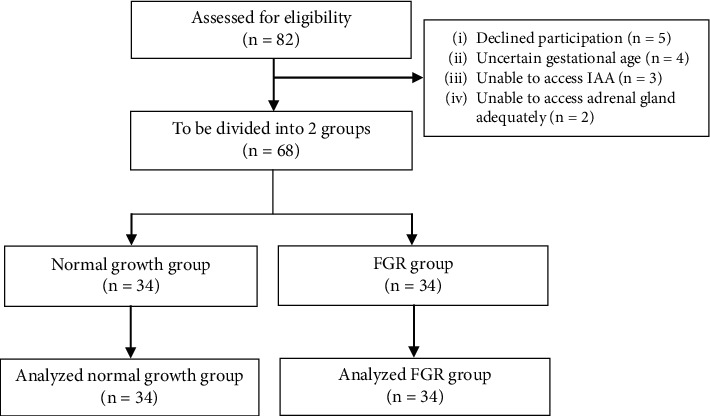
The study flow of the participants.

A total of 82 pregnant women were initially enrolled. Fourteen were excluded, including five who declined to participate, four with uncertain GA, three who could not be assessed for IAA, and two who could not have adequate adrenal gland sizes measured. The study flow is shown in Figure 3.

The baseline characteristics and neonatal outcomes are shown in Table 1. The mean maternal ages in the FGR and normal groups were 29.9 ± 4.4 and 30.0 ± 4.7 years, respectively. The GA at delivery for the FGR and normal groups was 35.9 ± 3.0 and 38.0 ± 1.8 weeks, respectively. The mean birth weights for the growth-restricted and normal infants were 1958.4 ± 590.6 and 3029.4 ± 406.1 g, respectively. The rates of NICU admission for the FGR and normal groups were 26.5% and 8.8%, respectively. The intrarater and interrater reliabilities were 0.87 (95% CI 0.65–0.95) and 0.84 (95% CI 0.59–0.94), respectively.

Table 2 compares the Doppler indices of the UA, MCA, and IAA for both groups. In the FGR group, UA PI, RI, and S/D were significantly higher, with *p* values of 0.005, 0.001, and 0.001, respectively. Additionally, the MCA PI, S/D, and CPR were significantly lower in the FGR group, with *p* values of 0.046, 0.039, and 0.004, respectively. Ten fetuses (29.4%) in the FGR group and none in the normal group had CPR below the 5th percentile (data not shown).

The IAA PSV of the FGR and the normal group were 14.9 ± 2.9 and 13.5 ± 2.0 cm/s, respectively. The IAA PSV in the FGR group was significantly higher than in the normal group with a mean difference of 1.4 cm/s (95% CI 0.27–2.56; *p* value 0.017). Additionally, the IAA PSV was higher in both early and late FGR compared to the normal group, although these differences were not statistically significant (*p* values 0.068 and 0.083, respectively). There were no statistical differences in the IAA PI, RI, and S/D between the FGR and normal group (*p* values 0.438, 0.441, and 0.658, respectively) (Table 2).

Table 3 compares the adrenal gland sizes between the two groups. The WAGV in the FGR and normal groups was 543.8 ± 342.0 and 586.9 ± 259.5 mm^3^, respectively. Despite this, both the C´WAGV and C´NV were significantly larger in the FGR group, with mean differences of 0.1 (95% CI 0.01–0.15) and 0.1 (95% CI 0.01–0.13) and *p* values of 0.031 and 0.020, respectively. In the sensitivity analysis, both the C´WAGV and C´NV of the late FGR group were also significantly higher, with mean differences of 0.1 (95% CI 0.002–0.19) and 0.1 (95% CI 0.005–0.16) and *p* values of 0.045 and 0.038, respectively (Table 3).

## 4. Discussion

The Doppler study of the IAA in fetuses with growth restriction revealed a significant increase in PSV, while no changes were observed in the PI, RI, and S/D compared to those with normal growth. Additionally, both the corrected WAG volume and the corrected neocortex volume were significantly enlarged in FGR.

Previous studies using Doppler assessment of the MAA to evaluate adrenal vessel adaptation in FGR have shown inconsistent results [37–39]. In our study, we observed a high IAA PSV in FGR, while the IAA PI, RI, and S/D remained unchanged. A high IAA PSV in FGR indicates possible blood flow redistribution to the adrenal gland. This could be a compensatory mechanism where the reduced blood volume from placental insufficiency is offset by increased blood flow from the numerous vessels supplying the adrenal glands. Similar observations have been reported in animal studies, which have demonstrated an increase in blood volume to the adrenal gland in conditions of chronic hypoxia and acidosis [16, 40, 41]. The arterial blood supply to the fetal adrenal glands is complex, primarily consisting of a network of arterioles within the adrenal cortex [21, 27]. As such, changes in the resistance of adrenal arteries in FGR are difficult to detect. Although the IAA PSV showed no significant changes in either the early or late FGR groups compared to the normal group, there was a notable trend towards an increase in the FGR group. This could potentially be attributed to the small sample sizes of these groups, which might have limited the statistical power to detect significant differences. Contrasting with our findings, Xu et al. reported that changes in IAA PI could be an early indicator of blood redistribution in FGR, prior to the brain-sparing effect [42]. However, our observations indicated that despite the brain-sparing effect in FGR, there were no significant changes in the IAA PI.

We suggest that the IAA is preferred for investigating fetal adrenal vessel adaptation as it primarily supplies the adrenal neocortex [26]. Additionally, the IAA is readily identifiable due to its origin at the trunk of the renal artery [27, 33].

Theoretically, hyperplasia and angiogenesis in the fetal adrenal cortex occur primarily in the neocortex zone [21–24, 43], indicating that fetoplacental adaptation in FGR might predominantly affect this zone. In contrast, the FZ undergoes rapid growth in the early trimester, followed by extensive apoptosis and scant mitotic figures in the later stages of gestation [21]. Therefore, changes in neocortex size significantly affect the overall size of the adrenal gland.

This study identified an increase in fetal WAG volume and neocortex volume, as evidenced by the significant enlargement of C´WAGV and C´NV. To our knowledge, this is the first study to compare fetal neocortex volume between FGR and normal groups. The exact mechanism of this enlargement in FGR remains unclear but may be attributed to blood flow redistribution from the placenta to the adrenal gland. Another plausible explanation is the increased cortisol synthesis in the neocortex via the HPA axis [20–22, 29, 42, 44]. Previous animal studies have shown elevated cortisol levels in cases of chronic placental insufficiency [16]. Additionally, fetal stress stimulates the release of placental corticotropin-releasing hormone (CRH), which positively influences ACTH receptor activity in the adrenal neocortex [21, 22, 26, 45]. Correspondingly, Farzad Mohajeri et al. reported an increase in corrected adrenal gland volume in FGR, which aligns with our findings [46]. Similarly, the study by Kaya and Polat demonstrated greater length and width of the adrenal cortex (akin to the neocortex) in FGR, while the width of the medulla (akin to the FZ) was smaller [47]. Heese et al. also noted a larger width of the fetal adrenal cortex and a higher width ratio between the total adrenal gland and medulla in FGR [28]. Furthermore, Hendem et al. reported that both adrenal gland volume and adrenal blood flow were increased in FGR [39].

The timing of the ultrasonographic evaluation of the fetal adrenal gland is crucial. In this study, five fetuses between 24 and 26 weeks of GA were excluded due to the inability to perform measurements. Both the IAA and the adrenal gland were difficult to identify in early gestation. Based on this evidence, we suggest that adrenal gland and IAA Doppler studies should be conducted after 27 weeks of GA. This recommendation is supported by Rosenberg et al., who reported that the adrenal gland was recognizable by ultrasonography in only 12% of fetuses below 26 weeks of GA and in 90% of fetuses over 26 weeks of GA [48].

The strengths of this study include its multicenter design, the prenatal diagnosis of all FGR cases, and the confirmation of these diagnoses at birth through both birthweight and histopathological evidence of UPI. The IAA was clearly identified by two sonographers prior to measurement, with good intra- and interrater reliability. However, there are several limitations to this study. Firstly, the neocortex volume values were based on indirect measurement. Secondly, measuring the adrenal gland volume and conducting the IAA Doppler study require significant expertise and experience, which may limit the generalizability of the findings to tertiary centers. Lastly, the findings from the sensitivity analysis should be interpreted with caution due to the small number of cases involved. Therefore, further research through more comprehensive studies is needed to confirm these initial findings.

## 5. Conclusion

Both increased IAA PSV and enlarged volumes of the corrected WAG and neocortex were found in fetuses with FGR, suggesting significant adrenal gland adaptation in response to chronic intrauterine stress.

## Figures and Tables

**Table 1:** Baseline maternal characteristics and neonatal outcomes.

**Variable**	**Normal growth**	**Fetal growth restriction**
	**(** **N** = 34**)**	**(** **N** = 34**)**
*Maternal characteristics*		
Age (years)	30.0 ± 4.7	29.9 ± 4.4
Primigravida	12 (35.3)	19 (55.9)
Prepregnancy BMI (kg/m^2^)	23.5 ± 4.6	20.9 ± 3.7
GA at the time of examination (weeks)	32.7 ± 2.5	32.9 ± 3.1
Delivery route		
Normal labor	20 (58.8)	14 (41.1)
Cesarean section	12 (35.3)	20 (58.8)
Vacuum extraction	2 (5.8)	0 (0)
*Neonatal outcomes*		
GA at delivery (weeks)	38.0 ± 1.8	35.9 ± 3.0
Male sex	16 (47.1)	11 (32.4)
Birth weight (grams)	3029.4 ± 406.1	1958.4 ± 590.6
Fetal distress	0 (0)	8 (23.5)
APGAR score at 1 m <7	0 (0)	7 (20.5)
Capillary blood gas pH	NA	7.4 ± 0.1
Respiratory distress	5 (14.7)	11 (32.4)
Early neonatal sepsis	0 (0)	5 (14.7)
Early neonatal jaundice	9 (26.5)	14 (41.2)
Polycythemia	0 (0)	3 (8.8)
Neonatal intensive care unit admission	3 (8.8)	9 (26.5)
Length of hospital stay (days)	4.7 ± 4.7	13.9 ± 14.1

*Note:* Values are presented as mean ± standard deviation for continuous data and number (%) for categorical data.

Abbreviations: BMI, body mass index; GA, gestational age; NA, not available.

**Table 2:** Comparison of Doppler indices of the umbilical artery, middle cerebral artery, and inferior adrenal artery between fetal growth restriction and normal groups.

**Doppler indices**	**Normal growth (** **N** = 34**)**	**Fetal growth restriction**			**Mean difference** ^a^ ** (95% CI)** ^d^	**p** ** value**	**Mean difference** ^b^ ** (95% CI)** ^d^	**p** ** value**	**Mean difference** ^c^ ** (95% CI)** ^d^	**p** ** value**
		**Total (** **N** = 34**)**	**Early (** **N** = 14**)**	**Late (** **N** = 20**)**						
Umbilical artery										
UA PSV (cm/s)	43.5 ± 9.8	41.9 ± 9.2	40.2 ± 6.1	43.0 ± 10.9	−1.7 (−6.36 to 2.92)	0.462	−3.1 (−10.45 to 4.22)	0.400	−0.7 (−6.73 to 4.41)	0.829
UA PI	1.0 ± 0.1	1.2 ± 0.3	1.3 ± 0.4	1.1 ± 0.3	0.2 (0.06–0.29)	0.005	0.2 (0.02–0.395)	0.030	0.1 (−0.02 to 0.29)	0.090
UA RI	0.6 ± 0.1	0.7 ± 0.1	0.8 ± 0.1	0.7 ± 0.1	0.1 (0.04–0.14)	0.001	0.1 (−0.008 to 0.15)	0.077	0.1 (0.02–0.16)	0.009
UA S/D	2.9 ± 2.9	3.8 ± 1.6	4.7 ± 2.2	3.2 ± 0.7	1.0 (0.41–1.52)	0.001	1.3 (0.42–2.19)	0.004	0.6 (−0.08 to 1.38)	0.081
Middle cerebral artery										
MCA PSV (cm/s)	50.5 ± 10.3	49.1 ± 13.1	43.9 ± 14.2	52.7 ± 11.4	−1.6 (−7.09 to 3.87)	0.560	−3.0 (−11.72 to 5.70)	0.492	−0.1 (−7.31 to 7.09)	0.975
MCA PI	1.8 ± 0.4	1.6 ± 0.4	1.7 ± 0.4	1.6 ± 0.3	−0.2 (−0.35 to −0.004)	0.046	−0.3 (−0.55 to −0.003)	0.048	−0.1 (−0.34 to 0.11)	0.306
MCA RI	0.8 ± 0.1	0.8 ± 0.1	0.8 ± 0.1	0.8 ± 0.1	−0.04 (−0.07 to 0.00)	0.064	−0.1 (−0.11 to 0.02)	0.136	−0.03 (−0.08 to 0.02)	0.222
MCA S/D	7.0 ± 6.6	4.6 ± 1.5	4.5 ± 1.4	4.7 ± 1.7	−2.4 (−4.67 to −0.12)	0.039	−5.0 (−8.47 to −1.43)	0.007	−0.8 (−3.69 to 2.14)	0.596
CPR	1.8 ± 0.4	1.5 ± 0.5	1.3 ± 0.5	1.5 ± 0.5	−0.3 (−0.11 to −0.55)	0.004	−0.5 (−0.79 to −0.11)	0.011	−0.2 (−0.53 to 0.03)	0.087
Inferior adrenal artery										
IAA PSV (cm/s)	13.5 ± 2.0	14.9 ± 2.9	14.1 ± 2.8	15.4 ± 2.8	1.4 (0.27–2.56)	0.017	1.7 (−0.13 to 3.51)	0.068	1.3 (−0.18 to 2.83)	0.083
IAA PI	1.1 ± 0.2	1.0 ± 0.2	1.0 ± 0.2	1.1 ± 0.2	−0.04 (−0.13 to 0.06)	0.438	−0.1 (−0.26 to 0.03)	0.121	−0.01 (−0.10 to 0.13)	0.805
IAA RI	0.6 ± 0.1	0.6 ± 0.1	0.6 ± 0.1	0.6 ± 0.1	−0.01 (−0.05 to 0.02)	0.441	−0.02 (−0.08 to 0.03)	0.319	−0.01 (−0.05 to 0.04)	0.833
IAA S/D	2.8 ± 0.5	2.8 ± 0.9	2.8 ± 1.1	2.7 ± 0.7	−0.1 (−0.43 to 0.27)	0.658	−0.1 (−0.65 to 0.45)	0.714	−0.1 (−0.53 to 0.39)	0.762

*Note:* Continuous data is presented as mean ± standard deviation.

Abbreviations: CI, confidence interval; CPR, cerebroplacental ratio; FGR, fetal growth restriction; IAA, inferior adrenal artery; MCA, middle cerebral artery; PI, pulsatility index; PSV, peak systolic velocity; RI, resistance index; SD, standard deviation; S/D, systolic/diastolic; UA, umbilical artery.

^a^Mean difference: total FGR versus normal growth.

^b^Mean difference: early FGR versus normal growth.

^c^Mean difference: late FGR versus normal growth.

^d^Mean differences and 95% CIs were adjusted by gestational age at the time of examination.

**Table 3:** Comparison of adrenal gland size between fetal growth restriction and normal groups.

**Adrenal gland sizes**	**Normal growth (** **N** = 34**)**	**Fetal growth restriction**			**Mean difference** ^a^ ** (95% CI)** ^d^	**p** ** value**	**Mean difference** ^b^ ** (95% CI)** ^d^	**p** ** value**	**Mean difference** ^c^ ** (95% CI)** ^d^	**p** ** value**
		**Total (** **N** = 34**)**	**Early (** **N** = 14**)**	**Late (** **N** = 20**)**						
WAG (mm^3^)	586.9 ± 259.5	543.8 ± 342.0	388.6 ± 171.1	652.4 ± 391.1	−51.3 (−185.48 to 82.82)	0.448	−42.9 (−255.48 to 169.76)	0.689	38.8 (−214.58 to 136.97)	0.661
C´WAGV	0.3 ± 0.1	0.4 ± 0.2	0.4 ± 0.2	0.4 ± 0.2	0.1 (0.01–0.15)	0.031	0.1 (−0.06 to 0.16)	0.358	0.1 (0.002–0.19)	0.045
FZV (mm^3^)	93.4 ± 49.9	84.4 ± 76.2	49.1 ± 28.3	109.1 ± 82.2	−10.9 (−48.76 to 27.00)	0.568	−9.1 (−69.29 to 50.94)	0.761	−7.9 (−57.57 to 41.82)	0.753
C´FZV	0.1 ± 0.02	0.1 ± 0.1	0.1 ± 0.01	0.1 ± 0.1	0.01 (−0.01 to 0.03)	0.418	−0.001 (−0.03 to 0.03)	0.941	0.01 (−0.01 to 0.04)	0.264
NV (mm^3^)	493.5 ± 219.1	459.4 ± 252.6	339.5 ± 155.9	543.3 ± 276.0	−40.5 (−145.15 to 64.25)	0.443	−33.7 (−199.57 to 132.19)	0.686	−30.9 (−168.07 to 106.20)	0.654
C´NV	0.2 ± 0.1	0.3 ± 0.1	0.3 ± 0.2	0.3 ± 0.1	0.1 (0.01–0.13)	0.020	0.1 (−0.04 to 0.15)	0.262	0.1 (0.005–0.16)	0.038

*Note:* Continuous data is presented as mean ± standard deviation.

Abbreviations: FZ, fetal zone; FZV, fetal zone volume; NV, neocortex volume; SD, standard deviation; WAG, whole adrenal gland; C´FZV, corrected fetal zone volume; C´NV, corrected neocortex volume; C´WAGV, corrected whole adrenal gland volume.

^a^Mean difference: total FGR versus normal growth.

^b^Mean difference: early FGR versus normal growth.

^c^Mean difference: late FGR versus normal growth.

^d^Mean differences and 95% CIs were adjusted by gestational age at the time of examination.

## Data Availability

The data and materials that support the findings of this study are available from the corresponding author, Chatuporn Duangkum, upon reasonable request.
